# Application of Natural and Calcined Oyster Shell Powders to Improve Latosol and Manage Nitrogen Leaching

**DOI:** 10.3390/ijerph20053919

**Published:** 2023-02-22

**Authors:** Xiaofei Yang, Kexing Liu, Yanmei Wen, Yongxiang Huang, Chao Zheng

**Affiliations:** 1 Faculty of Chemistry and Environmental Science, Guangdong Ocean University, Zhanjiang 524088, China; 2 College of Natural Resources and Environment, South China Agricultural University, Guangzhou 510642, China; 3 College of Coastal Agricultural Sciences, Guangdong Ocean University, Zhanjiang 524088, China; 4 South China Branch of National Saline-Alkali Tolerant Rice Technology Innovation Center Zhanjiang, Zhanjiang 524088, China

**Keywords:** oyster shell powder, calcination, latosol, soil acidification, pores, nitrate nitrogen, ammonium nitrogen, calcium leaching

## Abstract

Excessive N fertilizer application has aggravated soil acidification and loss of N. Although oyster shell powder (OSP) can improve acidic soil, few studies have investigated its ability to retain soil N. Here, the physicochemical properties of latosol after adding OSP and calcined OSP (COSP) and the dynamic leaching patterns of ammonium N (NH_4_^+^-N), nitrate N (NO_3_^−^-N), and Ca in seepage, were examined through indoor culture and intermittent soil column simulation experiments. Various types of N fertilizer were optimized through the application of 200 mg/kg of N, urea (N 200 mg/kg) was the control treatment (CK), and OSP and COSPs prepared at four calcination temperatures—500, 600, 700, and 800 °C—were added to the latosol for cultivation and leaching experiments. Under various N application conditions, the total leached N from the soil followed ammonium nitrate > ammonium chloride > urea. The OSP and COSPs had a urea adsorption rate of 81.09–91.29%, and the maximum reduction in cumulative soil inorganic N leached was 18.17%. The ability of COSPs to inhibit and control N leaching improved with increasing calcination temperature. Applying OSP and COSPs increased soil pH, soil organic matter, total N, NO_3_^−^-N, exchangeable Ca content, and cation exchange capacity. Although all soil enzyme activities related to N transformation decreased, the soil NH_4_^+^-N content remained unchanged. The strong adsorption capacities for NH_4_^+^-N by OSP and COSPs reduced the inorganic N leaching, mitigating the risk of groundwater contamination.

## 1. Introduction

Soil acidification is one of the main manifestations of soil degradation on arable land in South China; causing damages such as soil crusting and nutrient loss, inducing environmental problems such as water eutrophication and groundwater contamination, and limiting agricultural production. Global use of N fertilizers is increasing, and their excessive application has become an important factor that limits the fertility and agricultural production of arable land with acidic soils [1,2,3]. Due to soil acidification, the acidifying metal ions Al^3+^, Fe^2+^, and Mn^2+^, as well as protons (H^+^) are increased, and the alkaline and earth alkaine ions Ca^2+^, Mg^2+^, and K^+^, which are plant nutrients and buffer substances, are replaced. Consequently, Ca ions are easily leached during heavy tropical-subtropical rainfalls [4,5]. Currently, the application of modifiers (such as lime) is the main method for treating soil acidification and improving the utilization rate of N fertilizers. Guddisa et al. [6] reported that applications of lime increased soil pH, exchangeable Ca, and total N, but reduced exchangeable aluminum. Meena and Prakasha [7] and Mehnaz et al. [8] applied lime to acidic soils and noted that doing so not only adjusted and improved soil acidity but also increased crop yields and incomes.

Calcium carbonate (CaCO_3_) is a mineral with extensive natural existence and can be categorized as biological or mineral depending on the source. Compared with mineral-source CaCO_3_ such as lime, bio-source CaCO_3_ such as oyster shell powder (OSP) has a short formation cycle and is environmentally friendly and renewable. Therefore, OSP has received widespread attention for its application in improving acidic soils in recent years. The main component of oyster shells is CaCO_3_, which is slightly soluble in water, and alkaline, and can effectively remedy soil acidification [9] and improve soils that lack lime materials [10]. Yan [11] applied OSP to acidic yellow clayey fields and increased soil pH by 0.8. After adding 1 wt.% OSP or COSP to soils with an initial pH of approximately 5, Moon et al. [12] successfully increased soil pH to 7.

High-temperature calcination partially converts CaCO_3_ in OSP to easily soluble calcium oxide (CaO) and significantly increases the surface microporous structure [13,14]. Considering that calcined OSP (COSP) has the effects of improving soil acidity, increasing soil permeability, increasing water and fertilizer retention capacities, and supplementing Ca in the soil, it is an extremely promising environmentally friendly biomass soil conditioner. Xu et al. [15] and Li et al. [16] found that improvements in soil acidification were enhanced with additional amounts of applied COSP, leading to a soil pH of 0.29–1.39. Although the ability of COSP to improve soil acidification at various application rates has been generally recognized, its improvement abilities at different calcination temperatures and the related mechanism remain uncertain.

Some studies have concluded that soil acidification increases the positive charge of soil colloids, reduces the adsorption of base cations under the action of static electricity, and causes nutrients to easily leach during precipitation. Consequently, acidified soils have low nutrient content [17]. Applying alkaline OSP to soils subjected to the long-term application of chemical fertilizers can increase the base saturation and improve soil crusting and acidification, thus effectively increasing crop yields. Meanwhile, Jay et al. [18] and Lee et al. [19] reported that alkaline OSP has a certain adsorption effect on nutrients because of the abundant natural pores and negatively charged exchanger sites distributed on its surface, which act as ideal carriers for nutrient attachment.

The effectiveness of using conditioners to improve N nutrient availability in acidic soils is affected not only by chemical properties but also by microorganisms involved in the N cycling in the soil. According to Yun et al. [20], adding OSP to acidic mine soils increased soil microbial activities and inorganic N concentrations. However, large amounts of NH_4_^⁺^ were retained in the soil for a relatively long time period. Furthermore, the use of OSP in biochar- or polymer-treated soils revealed that it had significant short-term effects on soil N mineralization and acceleration of N turnover [21]. Most of these studies focused on the transformation of organic N to inorganic N in the soil. However, the mechanism by which applied inorganic conditioners improve acidic soils and N availability has not been thoroughly examined, which makes it crucial for related studies to be conducted.

Oyster shells are a form of solid shellfish waste generated in high volumes in coastal areas. They are alkaline and acquire excellent structure and strong adsorption capacities after thermal modification [22], meaning they can alleviate soil acidification arising from excessive fertilization and replenish exchangeable Ca and Mg contents in soils. The long-term application of lime conditioners alone causes soil crusting and nutrient disorders.

In this study, indoor cultures and leaching experiments were used to examine the effects of OSP and COSP prepared at various calcination temperatures on the physicochemical properties of latosol, enzyme activities related to N conversion, and N and Ca leaching. The aim was to provide a scientific basis for managing acidic soils, improving the quality of arable land, and promoting increased crop yields, thus addressing the governance of acidified arable land widely distributed in Southern China, the country’s main grain producing region. We hypothesized that OSP and COSP in latosol would increase soil pH and Ca content while reducing the leaching of NH_4_^+^-N and NO_3_^−^-N, making OSP and COSP excellent alternatives to lime materials.

## 2. Materials and Methods

### 2.1. Experimental Materials

The soil samples used for the culture and leaching experiments were acquired from paddy fields on the Leizhou Peninsula in Zhanjiang City, China (1100 insula, 2000 insula). The area has a tropical monsoon climate with an annual average temperature and precipitation of 22.5 °C and 1494.3 mm, respectively. The soil samples were surface latosol (0–20 cm) developed from basalt, which has a clay texture and is also known as ferralsol [23]. After the acquisition, the soil samples were transferred to the Institute of Agricultural Biotechnology, Guangdong Ocean University (110°30′07″ E, 21°14′85″ N), evenly mixed, and air-dried under natural conditions. They were then passed through a 1 mm sieve before being stored at 25 °C for later use. The latosol contained 27.65 mg/kg of alkali-hydrolyzed N, 13.84 mg/kg of available P, and 254.42 mg/kg of available potassium, measured using the methods discussed in Section 2.4. Its other basic physicochemical properties, shown in Table 1, are characterized by a marked lack of N and P and poor fertility.

The OSP was similar to that used in our previous study [24]. The oyster shells were collected and washed with a large amount of water until neutrality was reached and then dried and pulverized in OSP with a particle size of 0.15 mm (CaCO_3_ content = 96.88%). A ceramic fiber muffle furnace (TMF-36-10TP, Shanghai Zhetu Scientific Instrument Co., Ltd., Shanghai, China) was used to calcine the OSP at various temperatures—500, 600, 700, and 800 °C—for 2 h at a heating rate of 5 °C/min to prepare four types of COSP: 5COSP, 6COSP, 7COSP, and 8COSP, respectively. The calcination temperatures were selected because OSP undergoes severe weight loss at 600–800 °C, during which compositional and structural changes occur [25]. The pH of the OSP was greatly improved after calcination. The pH values of OSP, 5COSP, 6COSP, 7COSP, and 8COSP are 8.76, 9.97, 11.91, 13.15, and 13.22, respectively. N fertilizers used in the experiment were urea (46% N content), ammonium chloride (26% N content), and ammonium nitrate (35% N content), purchased from Guangzhou Huada Chemical Reagents Co., Ltd. (Guangzhou, China). Distilled water was used in the experiments, and the reagents were all analytically pure.

### 2.2. Leaching Simulation Device

The setup for the leaching simulations used in the current study was similar to that used by Nguyen et al. [5], but with slight modifications. The soil leaching column was made of plexiglass with an inner diameter of 6.5 cm, a height of 35 cm, and a cross-sectional area of 227.5 cm^2^. A valve was installed at the base of the column to control the leaching rate. In addition, 150 g of quartz sand (particle size 1–2 mm) was laid at the top and bottom of the column to form sand layers of approximately 3 cm thickness for filtering and to prevent soil disturbance. Between the two sand layers, there was a soil column of approximately 20 cm in height that comprised a mixture of N fertilizer-OSP/COSP soil (*w*/*w*).

The soil in the column was only moderately compacted to promote uniform leaching and ensure that the seepage during leaching did not flow along the column wall, and its bulk density was controlled at approximately 1.28 g/cm^3^. Small outlets were distributed at the column base. Below these and near the valve was a transparent hose (a drain pipe of 4 mm diameter) that was connected to a plastic sampling bottle for leachate collection. A cap on the sampling bottle minimized the evaporative loss of leachate.

### 2.3. Experimental Design

#### 2.3.1. Descriptive Experiments for the OSP and COSPs

##### Structure Analysis for the OSP and COSPs

The structural analysis of pre- and post-calcined OSP is of great significance to explore the effect of pore volume on N-storing capacity. During the experiments, small amounts of OSP and the four COSPs prepared at different calcination temperatures were used to observe the surface morphological characteristics under a scanning electron microscope (SEM) (Netzsch STA 2500, Shenzhen Taili Instruments (Shenzhen, China) and Apparatuses Co., Ltd., Bavaria, Germany). These were also measured using X-ray powder diffraction (XRD) (D8 ADVANCE, Bruker Corporation, Heidelberg, Germany), and their infrared spectra were measured using a Fourier transform infrared spectrometer (FTIR) (Nicolet iS10, Thermo Fisher Scientific, Waltham, MA, USA). Separately, a small amount of OSP was used to detect the characteristics of its thermodynamic changes with a thermogravimetric analyzer (TG) (TESCAN MIRA LMS, Beijing Yake Chenxu Technology Co., Ltd., Brno, Czech Republic).

##### N Adsorption Experiment for the OSP and COSPs

In this experiment, the N adsorption capacity of OSP and COSPs was quantified. A urea solution with an N concentration of 1 g/L was pre-configured and 20 mL of it was drawn into a 50-mL centrifuge tube. Next, 1 g of each OSP and four COSPs were added before the solutions were placed in a thermostatic oscillator to shake at 25 °C and at a constant speed of 150 r/min for 24 h. Specific-speed centrifugation was then performed at 4000 rpm for 5 min before the supernatant was retrieved to measure the N content. The residue was dried in an electrothermal draft drying oven at 50 °C until a constant weight was reached, and then 0.5 g of the mixture was weighed and wrapped with a 0.048 mm nylon mesh. The package was placed in a centrifuge tube containing 35 mL of distilled water before placing the tube in a thermostatic incubator at 25 °C. After 29 days of slow release, the supernatant was retrieved to measure the N content.

#### 2.3.2. Culture and Leaching Experiments

##### Culture Experiment for the OSP and COSPs

We studied the effects of OSP and COSPs on the physicochemical properties and enzyme activities of latosol, aiming to explore the mechanism by which they affected N leaching from the latosol. For this experiment, 100 g of latosol was weighed and placed in a brown jar. Urea with an N application level of 200 mg/kg was set as the control treatment. Urea at the same N application level and the OSP and four COSPs at the application level of 0.2 wt.% were mixed separately into the latosol and stirred well. After 25 mL of distilled water was added, the mouth of the jar was covered with plastic wrap punctured with small holes. The jar was then placed in a thermostatic incubator at 25 °C, with its water replenished every three days based on the weighing method to ensure that the soil moisture content was maintained at 25%. The jar was removed after 29 days of culturing, and the physicochemical properties and enzyme activities of the soil samples were determined.

##### Leaching Experiment for the OSP and COSPs

In this study, we explored the effects of OSP and COSPs on N leaching in latosol on the basis of optimizing N sources. Two soil column leaching experiments were conducted with 500 g of latosol weighed for each treatment. The first experiment was N fertilizer-free and acted as the control treatment. Urea, ammonium chloride, and ammonium nitrate were added separately to the latosol (*w*/*w*) as N sources to study the N leaching characteristics of the latosol with different types of N fertilizer. The selected N application level was 200 mg/kg because this is the N amount needed for conventional rice cultivation. In the second experiment, the N leaching characteristics of the three types of N fertilizer were compared. The selected N source was urea at 200 mg/kg. Next, OSP, 5COSP, 6COSP, 7COSP, and 8COSP were separately added (*w*/*w*) for culturing to further study the effects of the OSP and four COSPs on N leaching from latosol. The application level of the OSP and four COSPs for the experiment was 0.2 wt.%, which was based on the conventional application rate of lime to improve acidic soils.

**Figure 1:**
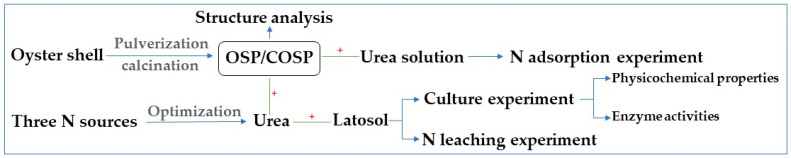
Flow chart graph of the experiment. “+” indicates that these experimental materials are mixed together.

For both experiments, 200 mL of distilled water was added to each of the soil columns. The soil was allowed to reach equilibrium for 24 h under the condition of 40% of the field capacity. Leaching was performed every 7 days using 200 mL of distilled water. The N leaching experiments lasted 29 days. Simultaneously, we have added Figure 1 for illustration to describe the complex experimental design more clearly.

### 2.4. Analytical Items and Methods

In the experiments, the pH of soil samples was measured using a pH meter (PHS-3B, Electrical Scientific Instruments Co., Ltd., Shanghai, China) and based on a water-soil ratio of 2.5:1. The bulk density and mechanical composition of the samples were measured using the ring-knife method and hydrometry, respectively. Organic matter and total N were measured using the potassium dichromate volumetric and semi-micro Kjeldahl method, respectively. Alkali-hydrolyzed N, NO_3_^−^-N, and NH_4_^+^-N were determined using the alkaline hydrolysis diffusion method, phenol disulfonic acid colorimetry, and 2 mol/L KCl extraction-ultraviolet spectrophotometry, respectively. Available P was determined using the 0.5 mol/L NaHCO_3_ method, available potassium was determined using NH_4_OAc extraction-flame photometry, and exchangeable Ca was determined using ethylenediaminetetraacetic acid complexometric titration.

The barium chloride method was used to determine the cation exchange capacity and conductivity was measured using a conductivity meter based on a water-soil ratio of 5:1. Urease activities were measured using indophenol blue colorimetry and expressed as mg of NH_3_-N produced per gram of dry soil over 24 h. Phenol disulfonic acid colorimetry was used to measure nitrate reductase activities, which were expressed as mg of NO_3_^−^-N produced per gram of dry soil over 24 h. Nitrite reductase activities were measured using α-naphthylamine colorimetry and expressed as mg of NO_2_-N produced per gram of dry soil over 24 h. Ammonium iron (III) sulfate-1,10-phenanthroline colorimetry was used to measure hydroxylamine reduction activities, which were expressed in mg of NH_2_OH produced per gram of dry soil for 5 h. The pH values of OSP and four COSPs were measured using the same methods as for soil samples.

The total N of the water samples was determined using the potassium persulfate oxidation method, and the pH was measured with a pH meter. NO_3_^−^N was determined using ultraviolet spectrophotometry, and NH_4_^+^-N was determined using indophenol blue colorimetry. Ca was measured using EDTA complexometric titration.

### 2.5. Data Processing

The experimental data were processed using Microsoft Excel 2010 software, and significance analysis was performed using one-way analysis of variance (ANOVA) in SPSS 21.0 statistical software. Multiple comparisons were made using the least significant difference (LSD) test, with the significance level set to α = 0.05. Origin 2022 software (OriginLab Corporation, Northampton, MA, USA) was used to prepare the images.

The adsorption and release rates of urea by the OSP and four COSPs were calculated using Equations (1) and (2):

(1) $$\alpha_{i}=\frac{20-M_{i}}{20}\times 100\%\;$$

(2) $$\beta_{i}=\frac{W_{i}}{20-M_{i}}\times 100\%\;$$

where $\alpha_{i}$ is the urea N adsorption rate in water under treatment i, %; $\beta_{i}$ is the N release rate in water after urea adsorption during treatment i, %; $M_{i}$ is the N content in the supernatant after urea adsorption during treatment i, mg; $W_{i}$ is the N content in water after urea adsorption during treatment i; and 20 is the amount of urea (per N calculation), mg.

Equation (3) was used to calculate the cumulative leaching amounts of N and Ca:

(3) $$\gamma_{i}=\overset{5}{\underset{i=1}{\sum }}C_{ij}\times V_{ij}\;$$

where $\gamma_{i}$ is the cumulative leaching amount of NH_4_^+^-N, NO_3_^−^-N, or Ca (mg); $C_{ij}$ is the mass concentration of NH_4_^+^-N, NO_3_^−^-N, or Ca in the leachate of the jth leaching test of treatment i (mg/L); and $V_{ij}$ is the volume of leachate of the jth leaching test of treatment i (mL).

N loss rate through leaching was calculated using Equation (4):

(4) $$\delta_{i}=\frac{N_{i}-N_{0}}{100}\times 100\%\;$$

where $\delta_{i}$ is the N loss rate through leaching during treatment i, %; $N_{i}$ is the cumulative leaching amount of NH_4_^+^-N or NO_3_^−^-N during treatment i, mg; $N_{0}$ is the cumulative leaching amount of NH_4_^+^-N or NO_3_^−^-N under the fertilizer-free treatment, mg; and 100 is the amount of urea (per N calculation), mg.

## 3. Results

### 3.1. Structural Characteristics of the OSP and Four COSPs

**Figure 2:**
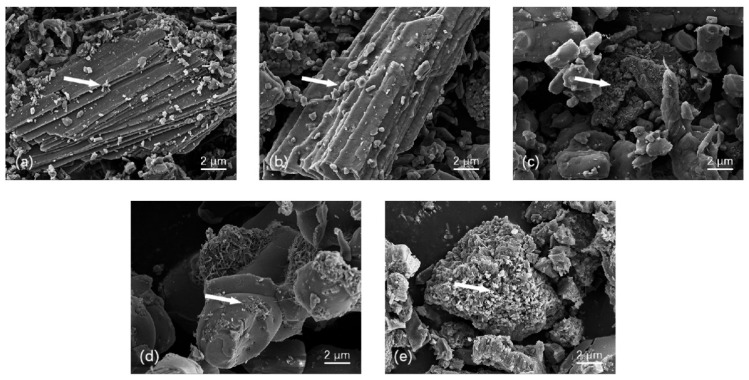
SEM images of pre- and post-calcined OSP. (**a**): Inactivated OSP; (**b**–**e**): 5COSP, 6COSP, 7COSP, and 8COSP (OSP after calcination for 2 h at 500, 600, 700, and 800 °C, respectively).

The inactivated OSP had uneven particle sizes, a smooth surface, and practically no pores, as shown in its SEM image in Figure 2a. These characteristics are not conducive to adsorption. The SEM images of the four COSPs after 2 h of calcination at various temperatures are shown in Figure 2b–e. The surfaces became more complex with increasing calcination temperatures. We also observed continuous increases in irregular micropores, lamellar gaps, and surface area of particles, resulting in structures that facilitated adsorption.

**Figure 3:**
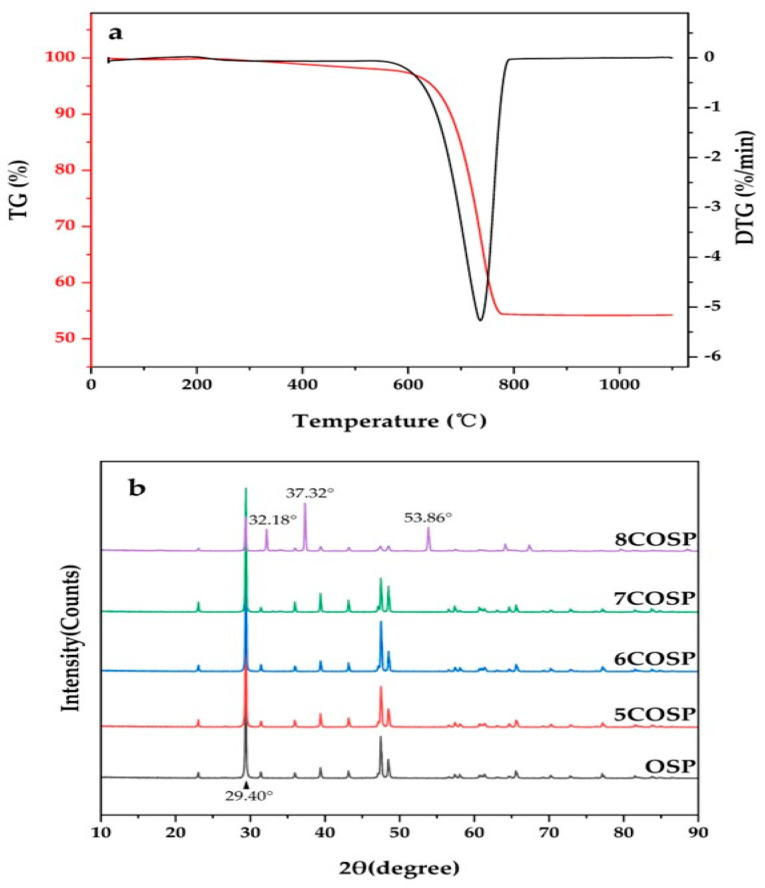
TG-DTA curves of the OSP and XRD patterns of pre- and post-calcined OSP. (**a**): TG-DTA curves of OSP; (**b**): XRD patterns of pre- and post-calcined OSP.

The thermogravimetric (TG) analysis results of the OSP are shown in Figure 3a. A simple endothermic process occurred at 0–682.5 °C, during which adsorbed water evaporated and molecular water, structural water, and a minute amount of organic matter were emitted, causing a small loss of mass. However, the rate of weight loss reached 45.75% at 682.5–763.7 °C and the speed of the weight loss rate was 5.27%/min at the peak temperature of 736.5 °C. The mass loss at this stage was mainly caused by the decomposition of CaCO_3_, which led to the release of CO_2_ (Figure 3b). The actual calcination temperatures varied slightly from the CaCO_3_ decomposition temperatures reflected by the TG-DTG curve because thermal decomposition was affected by various mechanisms such as thermal transfer, diffusion, and chemical reaction. Figure 2b also shows that the XRD patterns of 5–7COSP were highly similar to those of OSP, with the characteristic absorption peaks of CaCO_3_ displayed at 29.40°; however, 8COSP showed the characteristic absorption peaks of CaO at 32.18°, 37.32°, and 53.86°.

**Figure 4:**
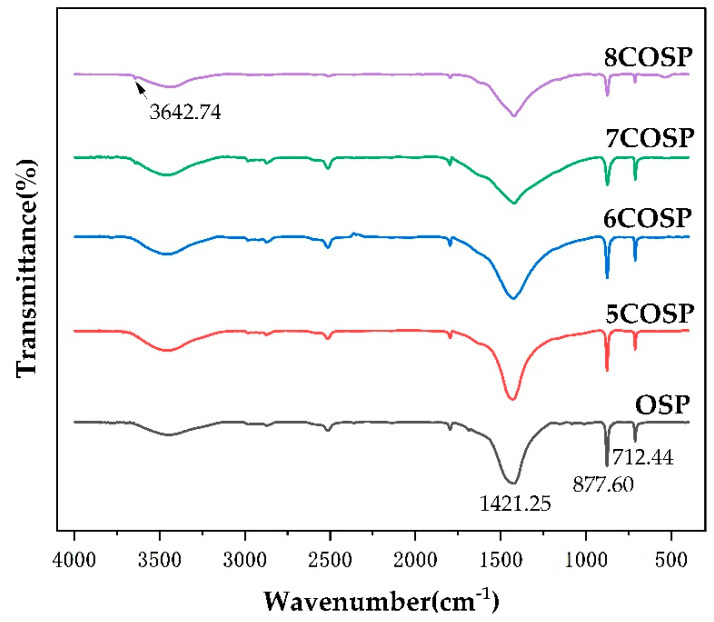
FTIR spectra of pre- and post-calcined OSP.

The absorption peak positions, vibration modes, and spectral bands of the FTIR spectra of the OSP and four COSPs are listed in Table 2. The width and position of the absorption peaks varied slightly because the OSP lattice structure was altered by calcination, which weakened the crystal field effect. The characteristic peaks of hydroxyl functional groups in OSP, 5COSP, 6COSP, 7COSP, and 8COSP treatments appeared at 3440.48, 3433.51, 3434.77, 3434.98, and 3431.03 cm^−1^, respectively. The FTIR spectra of the OSP and four COSPs are shown in Figure 4. The characteristic peaks of CaCO_3_ appeared at 712.44, 877.60, and 1421.25 cm^−1^ respectively, in OSP. It can be seen that the main component of the OSP was CaCO_3_, which remained basically unchanged when the calcination temperature was below 600 °C; only a little organic matter was emitted. However, CaCO_3_ began decomposing when the temperature rose above 600 °C. When the temperature was 700 to 800 °C, the COSPs comprised a mixture of CaCO_3_ and CaO, which is due to the characteristic peak of CaO at 3642.74 cm^−1^.

### 3.2. Adsorption Characteristics of Urea by the OSP and COSPs

**Figure 5:**
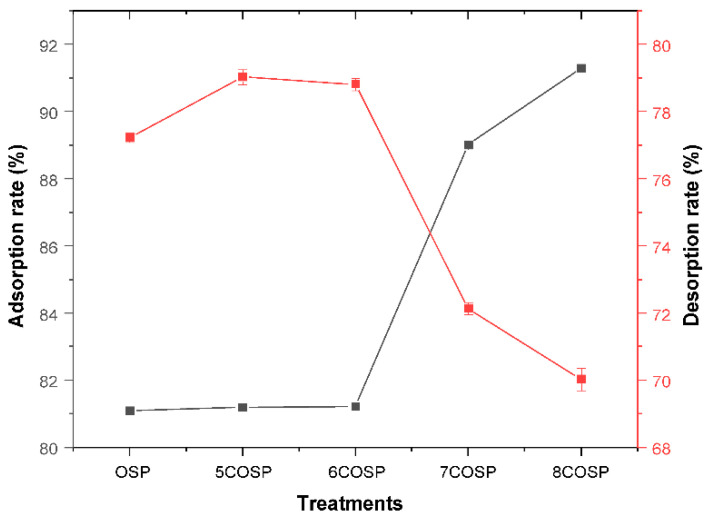
Urea adsorption and release rates under different treatments. OSP: 0.2 wt.% OSP; 5–8COSP: 0.2 wt.% OSP calcined at various temperatures; particle size of the OSP and four COSPs = 0.15 mm.

The relationship between calcination temperature and the adsorption rate of urea by OSP is reflected in Figure 5. With increasing calcination temperatures, the adsorption rate of urea in water by the COSPs rose threshold-like at calcination temperatures >600 °C and reached the maximum at 800 °C. At this time, the maximum adsorption amount and rate were 18.29 g and 91.29%, respectively. This might be because the micropores and gaps of the COSPs continued to grow with increasing temperatures, providing more adsorption sites for urea and expanding their adsorption capacities. After 29 days of treatment, 8COSP had the slowest (0.44 mg/day) and the lowest urea release rate (70.03%) among the treatments. For comparison, the urea adsorption rate by OSP was 80.09%, which was 11.2% lower than 8COSP; the release rate of urea by OSP was 77.22%, which was 7.19% higher than 8COSP. Overall, the adsorption and retention capacities of urea by OSP were weaker than those of COSPs. Thus, high-temperature calcination was an important way of improving N retention by OSP.

### 3.3. Impact of OSP and COSPs on the Physicochemical Properties of Latosol

Compared to CK treatment, the pH values after the OSP and COSP treatments increased significantly by 2 (OSP) and 2.10–2.22 (COSP), respectively (Table 3). The organic matter content also increased significantly by 7.54 and 6.17–13.10%, respectively, and total N content increased significantly by 13.24% and 14.71–26.47%, respectively. NO_3_^−^-N contents, exchangeable Ca contents, and cation exchange capacities increased significantly by 10.96% and −0.79–5.61%, 49.69% and 50.76–82.28%, and 117.04% and 19.76–83.97%, respectively.

These results indicated that the applications of the OSP and four COSPs effectively improved soil pH, organic matter, total N, exchangeable Ca content, and cation exchange capacity. However, their applications did not lead to any significant differences in soil NH_4_^+^-N content. Furthermore, no significant differences were observed between the 8COSP and CK treatments in terms of soil NO_3_^−^-N content. Soil pH, organic matter, total N, and exchangeable Ca content gradually increased with increasing calcination temperatures: when the temperature reached 800 °C, COSP had better improvement effects than OSP. However, soil NO_3_^−^-N content and cation exchange capacity gradually decreased with increasing calcination temperatures: when the temperature reached 800 °C, the improvement effects of COSP were inferior to those of OSP.

### 3.4. Impact of OSP and COSPs on Enzyme Activities in Latosol

**Figure 6:**
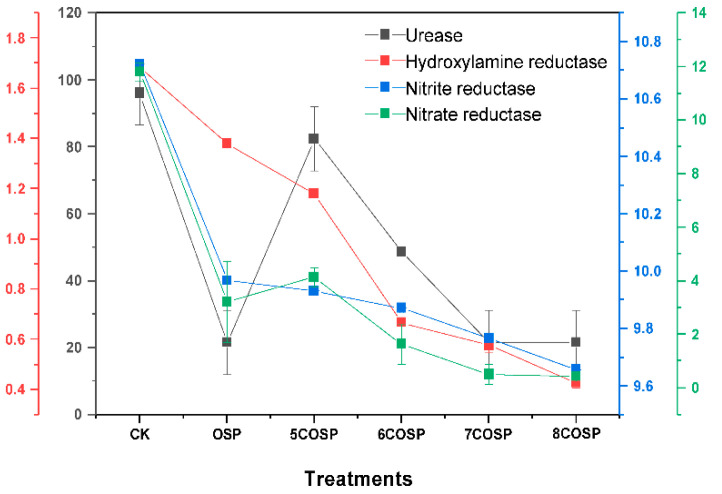
Enzyme activities of soils cultured with the treatments. CK: Urea treatment had a N application level of 200 mg/kg; OSP: 0.2 wt.% OSP; 5–8COSP: 0.2 wt.% OSP calcined at various temperatures; particle size of the OSP and four COSPs = 0.15 mm; the unit for soil urease, nitrate reductase, and nitrite reductase is mg·g^−1^·24 h^−1^; the unit for hydroxylamine reductase is mg·g^−1^·5 h^−1^.

The activities of soil urease, nitrate reductase, nitrite reductase, and hydroxylamine reductase were significantly affected by the OSP and four COSPs (Figure 6). The activities of soil enzymes related to N transformation decreased significantly with increasing calcination temperatures. Activities of urease, nitrate reductase, nitrite reductase, and hydroxylamine reductase treated with 8COSP decreased by 73.78%, 63.77%, 2.75%, and 89.58%, respectively.

Compared to OSP treatment, the application of 5COSP significantly increased soil urease and nitrate reductase activities by 2.81 and 1.29 times, respectively. However, after 8COSP treatment, the activities of nitrate reductase, nitrite reductase, and hydroxylamine reductase decreased by 86.60%, 3.12%, and 68.88%, respectively. Overall, the activities of soil urease, nitrate reductase, nitrite reductase, and hydroxylamine reductase decreased significantly by 14.09–77.47%, 72.77–96.35%, 7.04–9.94%, and 18.16–74.62%, respectively, after the OSP and COSP treatments, compared to CK treatment.

### 3.5. Impact of N Fertilizer Types on the Characteristics of NH_4_^+^-N and NO_3_^−^-N Leaching in Latosol

**Figure 7:**
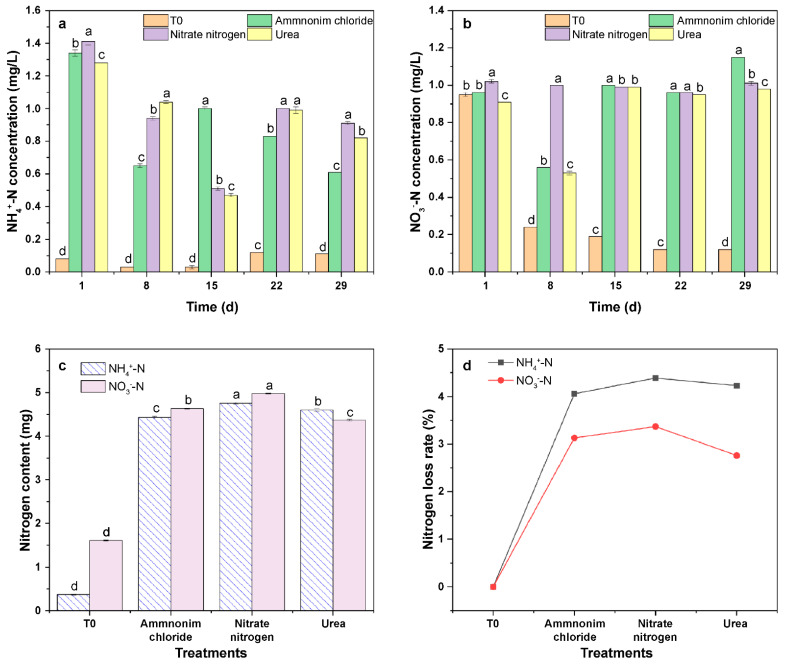
Variations in the (**a**,**b**) NH_4_^+^-N and NO_3_^−^-N concentrations, (**c**) cumulative leaching losses, and (**d**) leaching rates under the treatments. T0 treatment: Latosol without addition of fertilizer in the column; Ammonium chloride, Nitrate nitrogen, and Urea treatments: Latosol containing addition of fertilizer in the column; the N application levels of the urea, ammonium chloride, and ammonium nitrate treatments were similar at 200 mg/kg; the cumulative leaching losses of NH_4_^+^-N and NO_3_^−^-N under the fertilizer-free treatment were 0.37 and 1.61 mg, respectively; the different letters in the same column indicate that the differences were statistically significant at the *p* < 0.05 level nitrogen.

After treatments with ammonium chloride, ammonium nitrate, and urea, the NH_4_^+^-N concentrations in the leachate peaked at 1 day (1.34, 1.41, and 1.28 mg/L, respectively). The NO_3_^−^-N concentrations in leachate peaked at 29 days (1.15 mg/L), 1 day (1.02 mg/L), and 15 days (0.99 mg/L) for the three treatments, respectively (Figure 7a,b).

The cumulative amounts of NH_4_^+^-N in the leachate were in the order of ammonium nitrate > urea > ammonium chloride > T0 (Figure 7c), with those of the ammonium chloride, ammonium nitrate, and urea treatments being 11.10, 12.00, and 11.56 times higher than those of the T0 treatment, respectively. The cumulative amounts of NO_3_^−^-N in the leachate were of the order of ammonium nitrate > ammonium chloride > urea > T0, with those of the ammonium nitrate, ammonium chloride, and urea treatments being 1.87, 2.08, and 1.70 times higher than those of the T0 treatment, respectively. The NH_4_^+^-N loss rate of the urea treatment was 4.23%, which was 0.16% lower than the ammonium nitrate treatment and 0.17% higher than the ammonium chloride treatment. The NO_3_^−^-N loss rate was 2.76%, which was 0.37% and 0.61% lower than the ammonium chloride and ammonium nitrate treatments, respectively.

### 3.6. Impact of the OSP and COSPs on the N Leaching Characteristics of Latosol

#### 3.6.1. Impact of OSP and COSPs on the pH and Ca in Leachate

**Figure 8:**
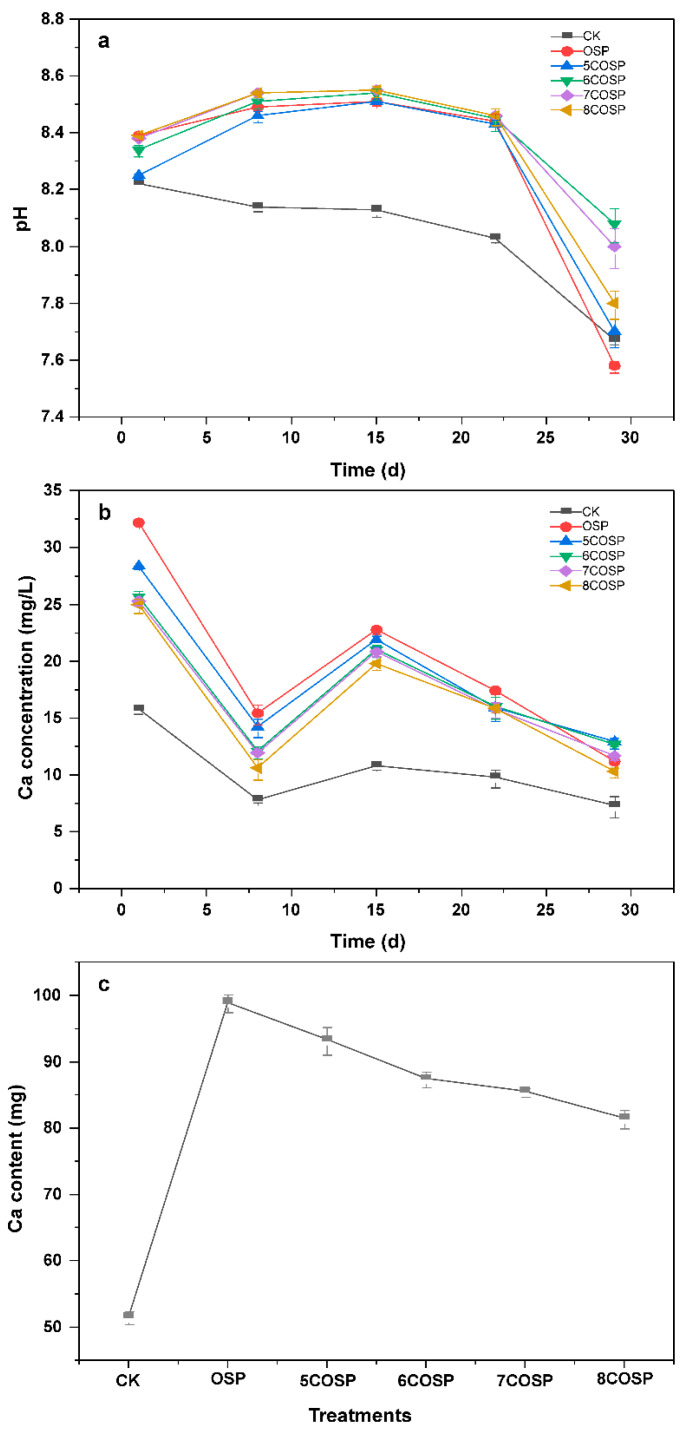
Variations in (**a**) pH and (**b**) Ca concentrations and (**c**) cumulative leaching concentrations under the treatments. CK: Urea treatment with N application level of 200 mg/kg; OSP: 0.2 wt.% OSP; 5–8COSP: 0.2 wt.% OSP calcined at various temperatures; particle size of the OSP and four COSPs = 0.15 mm.

With repeated leaching, the pH value of the leachate of the CK treatment continuously decreased, whereas that of the leachate of the OSP and COSP treatments exhibited a trend of initially increasing and then decreasing. The overall trend was the pH rising with increasing calcination temperatures (Figure 8a). Thus, the application of OSP and COSPs could significantly increase soil pH and alleviate soil acidification. This could be because the OSP and COSPs were alkaline (pH 8.76–13.22). After application to the soil, the Ca they contained dissolved in water and increased the base saturation of the soil, increasing the soil pH.

The results showed that with repeated leaching, the Ca leaching concentrations of all treatments presented a trend of first decreasing, then increasing to the highest concentration in 1 day, and finally decreasing (Figure 8b). The lowest concentration under the CK and 5COSP treatments appeared in 8 days, whereas that under the OSP and 6–8COSP treatments appeared in 29 days. The application of OSP and COSPs increased the soil Ca contents, and part of the Ca not adsorbed by the soil contributed to the cumulative Ca leaching amount through seepage. The cumulative leaching of soil Ca decreased significantly with increasing OSP calcination temperatures (*p* < 0.05).

#### 3.6.2. Impact of the OSP and COSPs on NH_4_^+^-N and NO_3_^−^-N in the Leachate

**Figure 9:**
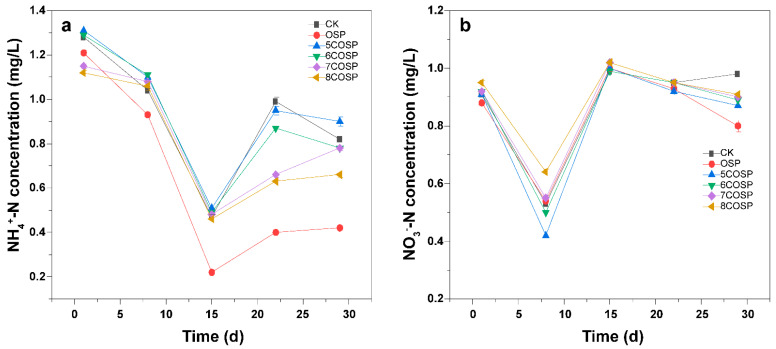
Variations in (**a**) NH_4_^+^-N and (**b**) NO_3_^−^-N concentrations of the leachate from the soil column under various treatments. CK: Urea treatment with N application level of 200 mg/kg; OSP: 0.2 wt.% OSP; 5–8COSP: 0.2 wt.% OSP calcined at various temperatures; particle size of the OSP and four COSPs = 0.15 mm.

During the culturing period, the NH_4_^+^-N and NO_3_^−^-N in the leachate decreased, increased, and then decreased again (Figure 9). The lowest NH_4_^+^-N and NO_3_^−^-N concentrations were recorded on days 15 and 8, respectively. In the early culturing stage, the NH_4_^+^-N concentration in the leachate of the treatments reached the peak value in 1 day, which was related to the texture and background N value of the soil tested. On days 8–29, the NH_4_^+^-N concentrations in the leachate of the 5–8COSP treatments were significantly higher than those of the OSP treatment. On day 29, the NO_3_^−^-N concentration in the leachate of the OSP treatment was significantly lower than the COSP treatments. This indicated that adding OSP effectively reduced the leaching of NH_4_^+^-N and NO_3_^−^-N, whereas calcined OSP increased the leaching of NH_4_^+^-N and NO_3_^−^-N.

The optimal period for the reduction of NH_4_^+^-N concentration by the treatments was 29 days: the concentrations were 0.42, 0.95, 0.87, 0.66, and 0.63 mg/L for OSP, 5COSP, 6COSP, 7COSP, and 8COSP, respectively. The treatments only had a significant effect in reducing the NO_3_^−^-N concentration in leachate after 29 days: the concentrations were 0.80, 0.87, 0.89, 0.90, and 0.91 mg/L for OSP, 5COSP, 6COSP, 7COSP, and 8COSP, respectively.

#### 3.6.3. Impact of the OSP and COSPs on the Leachate Volume

**Figure 10:**
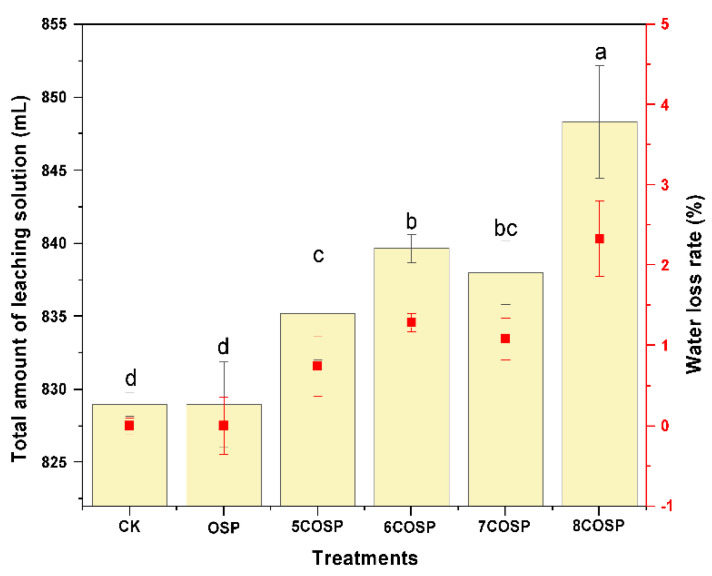
Cumulative volume and loss rate of leachate under the treatments. CK: Urea treatment with N application level of 200 mg/kg; OSP: 0.2 wt.% OSP; 5–8COSP: 0.2 wt.% OSP calcined at various temperatures; particle size of the OSP and four COSPs = 0.15 mm. The different letters in the same column indicate that the differences were statistically significant at the *p* < 0.05 level.

The leachate volume collected under the treatments was slightly different throughout the entire leaching process (Figure 10). When the leaching experiment ended, the cumulative leachate amount of the soil column under the OSP, 5COSP, 6COSP, 7COSP, and 8COSP treatments increased with increasing calcination temperature, reaching 829.00, 835.17, 839.67, 838.00, and 848.33 mL, respectively, with differences among the treatments being significant. The pore volume of OSP/COSP is increasing with calcination temperature, which would lead to increased water holding capacity and decreased water evaporation. The extra water needed to be added is reduced (reaching saturation), and the amount of water directly infiltrated is increased, resulting in an increase in leachate volume. Compared to CK treatment, the leachate volume of various COSP treatments increased significantly. However, the leachate volume of the OSP treatment was not significantly different from that of the CK treatment (*p* < 0.05), compared to which the leachate volumes of the 5COSP, 6COSP, 7COSP, and 8COSP treatments increased by 0.74%, 1.29%, 1.09%, and 2.33%, respectively. The experimental results showed that the application of appropriate amounts of COSPs increased soil cumulative infiltration, reducing the risk of Al-hydoxide covers of soil aggregates, that would be mobilized after de-acidification.

#### 3.6.4. Impact of OSP and COSPs on Cumulative N Leaching and Leaching Rate of Leachate

The OSP and COSP treatments had a certain restricting and controlling effect on NH_4_^+^-N and NO_3_^−^-N leaching in the soil (Table 4). The cumulative leaching amounts of NH_4_^+^-N and NO_3_^−^-N under the treatments were lower than those under the CK treatment by 0.07–1.63 mg, and the leaching rate decreased by 0.07–1.63%. The cumulative leaching amount and rate of NH_4_^+^-N and NO_3_^−^-N in leachate treated with OSP were obviously lower than those treated with COSPs. The lowest NH_4_^+^-N leaching rate under OSP was 2.82%, whereas that under COSP treatments was 3.56–4.40%. However, the NH_4_^+^-N leaching rate in soils increased with increasing COSP calcination temperatures.

The NO_3_^−^-N leaching rate of the OSP treatment was significantly lower than the CK treatment. However, the NO_3_^−^-N leaching rate increased significantly with increasing calcination temperatures of the COSPs. There were also significant differences between treatments (*p* < 0.05). N was leached in the form of NO_3_^−^-N and its cumulative leaching amount accounted for 48.39–56.53% of the total NH_4_^+^-N + NO_3_^−^-N leaching. The proportion of NO_3_^−^-N leaching gradually increased with increasing calcination temperatures of the COSPs, and it was significantly higher than NH_4_^+^-N when the calcination temperatures were above 700 °C.

## 4. Discussion

### 4.1. Impact of the OSP and COSPs on Latosol Acidification

After OSP was calcined, there was a significant improvement in the alkalescence of the resulting COSP. We applied OSP and COSPs to acidic soils separately and found that the soil pH adjusted by COSPs was 0.10–0.17 higher than that adjusted by OSP (Table 3). Ok et al. [26] also reported that after 30 d of cultivation, the acid-regulating effect on the soil by COSP was more significant than that of OSP, with the soil pH increasing by 4.2. The impact of OSP and COSPs on soil acidification manifested in two aspects. First, the hydroxyl functional groups in OSPs and COSPs, as shown in the FTIR spectrum in Figure 4, neutralized H^+^ in soils and increased soil pH. Second, soil base saturation increased because OSP and COSPs increased the exchangeable Ca content in soils (Table 3), thus increasing soil pH. However, the conditions for high-temperature calcination of OSP are stringent, causing the cost to increase correspondingly. Notably, the greenhouse effect caused by carbon dioxide produced after the calcination of oyster shell powder is also an environmental problem that cannot be ignored. The comparison between the CO_2_ release through calcination and the benefits intended by the application of COSP is worthy of further study. The economic and environmental costs of preparing OSP are lower than COSP, which may it more economically applicable. Furthermore, the restricting and controlling effects of OSP on soil NH_4_^+^-N leaching were better than those of COSP treatments (Table 4), indicating that OSP could be an ideal alternative to lime materials.

### 4.2. Impact of OSP and COSPs on N Leaching in Latosol

The main route of N loss from arable land is N leaching, which is heavily affected by fertilizer types [27,28,29]. The experiments in this study were conducted based on simulations using indoor soil columns. Three N fertilizer types were applied on the premise of a similar application amount. The results showed that ammonium nitrate was the most likely to be leached, followed by ammonium chloride, and finally urea (Figure 7). Previous studies have shown that slow-release N from urea leads to less NO_3_^−^-N leaching than fast-release N sources (ammonium nitrate) [30], which agreed with the findings of this study.

Although OSP (and especially COSP) is used primarily to improve acidic soils, there is a lack of studies on N leaching related to their applications. This study indicated that adding OSP in addition to urea (N level of 200 mg/kg) reduced the leaching amounts of NH_4_^+^-N, NO_3_^−^-N, and NH_4_^+^-N + NO_3_^−^-N by 30.65%, 5.03%, and 18.17%, respectively, compared with the non-OSP (CK) treatment. Studies have also shown that the application of OSP increased the amount of leaching of NO_3_^−^-N in soil by 2.1 times [21], which was higher than the amount of leaching of NO_3_^−^-N in this study. This was related to differences in soil textures and levels of N application—the clay content of the soil in that study was 22.6%, which was much lower than the 57.68% clay content of latosol in this study.

**Figure 11:**
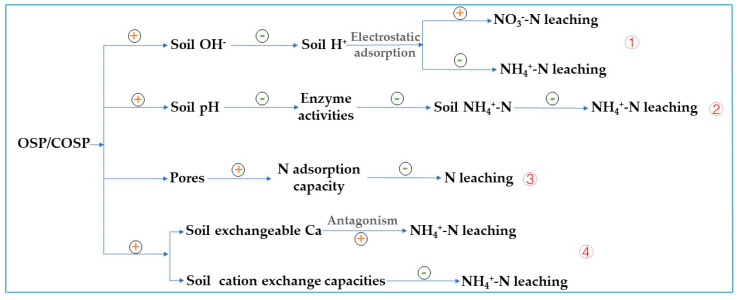
Four mechanisms by which pre- and post-calcined OSP affected N leaching from the latosol. We used four process hypotheses: (1) electrostatic adsorption of ammonium; (2) high pH reduces nitrate reducing enzymes; (3) increased pore volume enhances N-storing capacity; (4) soil cation exchange capacity decreasing ammonium leaching. “+” indicates an increase; “-” indicates a decrease.

There are four mechanisms by which pre- and post-calcined OSP affected N leaching from the latosol (Figure 11), and the results are jointly affected by one or more mechanisms.

(1) Electrostatic adsorption of ammonium. Alkaline OSP and COSPs increased OH^−^ in soil colloids, which effectively neutralized part of the soil H^+^ and enhanced its adsorption of soil cations, thus reducing soil NH_4_^+^-N and Ca^2+^ leaching. Figure 8c shows that the total Ca^2+^ in the soil leachate after COSP treatments was lower than that after OSP treatment. This was because the number of hydroxyl groups on the OSP surface increased after calcination.

(2) High pH reduces nitrate reducing enzymes. OSP affected soil N leaching was pH, which was 2–2.22 for OSP and COSPs. While increasing soil pH, their strong alkalinity reduced soil enzyme activities, slowed the rate of urea decomposition, and inhibited the conversion of NH_4_^+^-N to NO_3_^−^-N. After the OSP and COSP treatments. The soil pH increased significantly by 2 to 2.22 and the soil urease significantly by 14.09–77.47% (Table 3 and Figure 6). Wu et al. [31] noted that OSP application increased soil urease activities, which differed from this study and might be related to soil pH. Frankenberger et al. [32] reported that the optimal pH of soil urease was 6.5–7.0 and 8.8–9.0. However, the soil pH achieved after culturing with OSP and COSPs for 29 days was 7.47–7.64, which was not within the optimal pH of soil urease. The activities of soil denitrification enzymes (nitrate reductase, nitrite reductase, and hydroxylamine reductase) affect soil NH_4_^+^-N content, and there is a certain negative correlation between them. The soil denitrification enzyme activities of OSP and COSP treatments were significantly reduced (Figure 6), which may be similar to the effect of pH on soil urease. Furthermore, the pH after COSP treatments reached 13.22, creating a strong alkaline environment immediately after application in the soil and resulting in decreased activities of some soil enzymes.

(3) Increased pore volume enhances N-storing capacity. The OSP and COSP had certain adsorption capacities for NH_4_^+^-N and NO_3_^−^-N because their porous structures facilitated physical adsorption. There was also electrostatic adsorption of soil NH_4_^+^-N and Ca^2+^. SEM images in Figure 2 and the results in Figure 5 demonstrate that multiple pores were generated on the surface of the COSPs with increasing calcination temperatures, resulting in the adsorbed total N at the rates of 81.19–91.29%. Huh et al. [33] found that COSPs could condense and precipitate N by adsorption, with an average total N adsorption rate of 91%, which was similar to the results here.

(4) Soil cation exchange capacity decreases ammonium leaching. Soil exchangeable Ca content and cation exchange capacity increased through the addition of OSP and COSPs. The results in Table 3 indicate that after 29 days of adding OSP and COSPs to latosol and culturing, soil Ca^2+^ and cation exchange capacity increased by 49.69–84.28% and 19.76–117.04%, respectively. The addition of COSPs increased soil Ca^2+^ content more than adding OSP, which led to the weakening effect of COSPs to restrict and control NH_4_^+^-N leaching. This is because there was a certain level of antagonism between Ca^2+^ and NH_4_^+^-N. Tahir and Marschner [34] concluded that NH_4_^+^-N was more easily leached in soils with low cation exchange capacity. After adding COSPs, the soil cation exchange capacities decreased compared to adding OSP, resulting in a decrease in the soil adsorption of NH_4_^+^-N. In addition, OSP and COSPs applied to the soil also affect soil microorganisms, which may be a potential mechanism affecting soil N leaching and need to be considered in further research.

The results in Figure 10 show that adding 0.2 wt.% OSP or COSPs increased soil cumulative infiltration and improved soil permeability. According to Kim et al. [35], CaCO_3_ is the main component of OSP and promotes soil particle granulation, and affects the distribution of soil particle size distribution. In addition, the porous structure of OSP improves permeability when mixed with soil. Although COSPs and OSP shared similar main components, increases in the number of surface micropores on COSPs after calcination might account for their different effects on increasing soil infiltration. Another reason could be that soil organic matter content of COSP soil increased after the application compared to OSP (Table 3). There may be a strong association between Ca^2+^ and organic matter such as humic acid in soil [36], which can promote the formation of soil aggregates, make the soil loose, improve the water retention capacity, maintain the soil ecological environment suitable for the growth of dominant bacteria, promote the decomposition and transformation of carbon sources into organic matter, and thus increase the content of soil organic matter. Therefore, under the same amount of addition, the content of Ca^2+^ in COSP is higher than that in OSP, and the soil organic matter content is also increased. Liu et al. [37] found that soil cumulative infiltration increased with increasing soil organic matter content. Soils with additional organic matter contents contain more soil aggregates and higher porosity, which causes soil infiltration capacities to increase.

This study was based solely on simulations using indoor soil columns and cannot fully represent the actual situation of field planting. Furthermore, the test results might be affected by the application of OSPs and COSPs with different particle sizes and at various dosages, which we aim to explore in our further research.

## 5. Conclusions

In this study, urea N was less prone to leaching than ammonium nitrate and ammonium chloride when applied to latosol. The addition of OSP and COSPs effectively inhibited the acidification of latosol and N leaching (NH_4_^+^-N and NO_3_^−^-N). However, OSP treatment had better effects on soil N leaching, whereas COSP treatments were better at reducing soil acidification. Furthermore, the application of OSPs and COSPs effectively improved the physicochemical properties of soils. Significant increases in soil organic matter, total N, exchangeable Ca, and cation exchange capacity were observed with increasing calcination temperatures, although enzyme activities were reduced because of N transformation. Through comparison, it was concluded that either OSP or 8COSP (OSP calcined at 800 °C) should be selected for reducing the leaching of soil NH_4_^+^-N, NO_3_^−^-N, and Ca, and for improving soil pH and exchange performance.

## Figures and Tables

**Table 1:** Basic physicochemical properties of the tested soil.

Tested Material	Data	pH	Organic Matter (g/kg)	Total N (g/kg)	NO_3_^−^-N (mg/kg)	NH_4_^+^-N (mg/kg)	Exchangeable Ca (g/kg)
Latosol	Mean	4.85	4.94	0.26	6.97	0.96	12.88
	SE	0.03	0.49	0.02	0.04	0.00	0.10

Notes: Latosol contained 57.68% and 13.54% of clay and sandy content, respectively; its bulk density was 1.28 g/cm^3^, the cation exchange capacity was 10.39 cmol/kg, and the electrical conductivity was 70.37 µs/cm. SE = standard error, *n* = 3.

**Table 2:** Infrared spectrum assignment of pre- and post-calcined OSP.

Absorption Peak Position (cm^−1^)					Assignment
OSP	5COSP	6COSP	7COSP	8COSP	
*High-frequency region*					
3440.48	3433.51	3434.77	3434.98	3431.03	O-H symmetric stretching vibration
2872.59	2873.18	2873.16	2872.74	2872.65	-CH_2_- and -CH_3_ stretching vibrations
2509.87	2511.7	2511.7	2510.98	2511.91	-S-H symmetric stretching vibration
*Medium-frequency region*					
1796.49	1796.82	1797.22	1798.58	1796.5	C-O symmetric stretching vibration
1421.23	1428.36	1423.15	1420.95	1421.25	C-O asymmetric stretching vibration
874.6	877.12	875.85	874.85	877.6	CO_3_^2−^ out-of-plane bending vibration
712.9	712.43	712.26	711.81	712.44	CO_3_^2−^ in-plane bending vibration

**Table 3:** Physicochemical properties of soils cultured with the treatments.

Treatment	pH	Organic Matter (g/kg)	Total N (g/kg)	NO_3_^−^-N (mg/kg)	NH_4_^+^-N (mg/kg)	Ca (g/kg)	Cation Exchange Capacity (cmol/kg)
CK	5.47 ± 0.02 ^d^	13.13 ± 0.06 ^c^	0.68 ± 0.00 ^c^	15.15 ± 0.08 ^e^	1.97 ± 0.03 ^a^	17.69 ± 0.14 ^e^	9.92 ± 0.51 ^f^
OSP	7.47 ± 0.03 ^c^	14.12 ± 0.17 ^b^	0.77 ± 0.01 ^b^	16.81 ± 0.06 ^a^	1.98 ± 0.02 ^a^	26.48 ± 0.54 ^d^	21.53 ± 0.09 ^a^
5COSP	7.57 ± 0.02 ^b^	13.94 ± 0.15 ^b^	0.78 ± 0.03 ^b^	16.00 ± 0.02 ^b^	1.99 ± 0.01 ^a^	26.67 ± 0.23 ^d^	18.25 ± 0.47 ^b^
6COSP	7.58 ± 0.01 ^b^	13.96 ± 0.32 ^ab^	0.79 ± 0.01 ^ab^	15.48 ± 0.07 ^c^	1.99 ± 0.00 ^a^	28.59 ± 0.50 ^c^	11.88 ± 0.54 ^e^
7COSP	7.63 ± 0.02 ^a^	14.53 ± 0.24 ^a^	0.83 ± 0.03 ^a^	15.33 ± 0.07 ^d^	1.99 ± 0.00 ^a^	30.92 ± 0.26 ^b^	15.89 ± 0.41 ^d^
8COSP	7.64 ± 0.01 ^a^	14.85 ± 0.10 ^a^	0.86 ± 0.03 ^a^	15.03 ± 0.10 ^e^	2.00 ± 0.01 ^a^	32.60 ± 0.36 ^a^	17.45 ± 0.16 ^c^

CK: Urea treatment with N application level of 200 mg/kg; OSP: 0.2 wt.% OSP; 5–8COSP: 0.2 wt.% OSP calcined at various temperatures; particle size of the OSP and four COSPs = 0.15 mm; the different letters in the same column indicate that the differences were statistically significant at the *p* < 0.05 level.

**Table 4:** Cumulative leaching amount and rate of NH_4_^+^-N and NO_3_^−^-N under the treatments.

Treatment	Cumulative Leaching (mg)			NH_4_^+^-N/NH_4_^+^-N + NO_3_^−^-N (%)	NO_3_^−^-N/NH_4_^+^-N + NO_3_^−^-N (%)	Leaching Rate (%)		
	NH_4_^+^-N	NO_3_^−^-N	NH_4_^+^-N + NO_3_^−^-N			NH_4_^+^-N	NO_3_^−^-N	NH_4_^+^-N + NO_3_^−^-N
CK	4.60 ± 0.04 ^b^	4.37 ± 0.02 ^b^	8.97 ± 0.06 ^a^	51.31 ± 0.11 ^b^	48.69 ± 0.11 ^d^	4.16 ± 0.04 ^b^	2.76 ± 0.02 ^b^	6.99 ± 0.06 ^a^
OSP	3.19 ± 0.02 ^f^	4.15 ± 0.03 ^e^	7.34 ± 0.02 ^d^	43.47 ± 0.29 ^e^	56.53 ± 0.29 ^a^	2.82 ± 0.02 ^f^	2.54 ± 0.03 ^e^	5.36 ± 0.02 ^d^
5COSP	4.77 ± 0.05 ^a^	4.13 ± 0.00 ^e^	8.90 ± 0.05 ^a^	53.61 ± 0.23 ^a^	46.39 ± 0.23 ^e^	4.40 ± 0.05 ^a^	2.52 ± 0.00 ^e^	6.92 ± 0.05 ^a^
6COSP	4.53 ± 0.02 ^c^	4.23 ± 0.01 ^d^	8.78 ± 0.01 ^b^	51.61 ± 0.21 ^b^	48.39 ± 0.21 ^d^	4.16 ± 0.02 ^c^	2.64 ± 0.01 ^d^	6.80 ± 0.01 ^b^
7COSP	4.14 ± 0.03 ^d^	4.33 ± 0.01 ^c^	8.47 ± 0.03 ^c^	48.88 ± 0.21 ^c^	51.12 ± 0.21 ^c^	3.77 ± 0.03 ^d^	2.72 ± 0.01 ^c^	6.49 ± 0.03 ^c^
8COSP	3.93 ± 0.03 ^e^	4.47 ± 0.03 ^a^	8.40 ± 0.06 ^c^	46.75 ± 0.09 ^d^	53.25 ± 0.09 ^b^	3.56 ± 0.03 ^e^	2.86 ± 0.03 ^a^	6.42 ± 0.06 ^c^

CK: Urea treatment with N application level of 200 mg/kg, 500 g of latosol, the actual N application rate was 100 g (per N calculation); particle size of the OSP and four COSPs = 0.15 mm; the cumulative NH_4_^+^-N and NO_3_^−^-N leaching losses under the fertilizer-free treatment were 0.37 and 1.61 mg, respectively; the different letters in the same column indicate that the differences were statistically significant at the *p* < 0.05 level.

## Data Availability

All data are mentioned in the body of the manuscript, tables, and figures.
